# Analysis and Study of Diabetes Follow-Up Data Using a Data-Mining-Based Approach in New Urban Area of Urumqi, Xinjiang, China, 2016-2017

**DOI:** 10.1155/2018/7207151

**Published:** 2018-07-10

**Authors:** Yukai Li, Huling Li, Hua Yao

**Affiliations:** ^1^College of Public Health, Xinjiang Medical University, Urumqi 830011, China; ^2^Center of Health Management, The First Affiliated Hospital of Xinjiang Medical University, Urumqi, Xinjiang 830054, China

## Abstract

The focus of this study is the use of machine learning methods that combine feature selection and imbalanced process (SMOTE algorithm) to classify and predict diabetes follow-up control satisfaction data. After the feature selection and unbalanced process, diabetes follow-up data of the New Urban Area of Urumqi, Xinjiang, was used as input variables of support vector machine (SVM), decision tree, and integrated learning model (Adaboost and Bagging) for modeling and prediction. The experimental results show that Adaboost algorithm produces better classification results. For the test set, the G-mean was 94.65%, the area under the ROC curve (AUC) was 0.9817, and the important variables in the classification process, fasting blood glucose, age, and BMI were given. The performance of the decision tree model in the test set is relatively lower than that of the support vector machine and the ensemble learning model. The prediction results of these classification models are sufficient. Compared with a single classifier, ensemble learning algorithms show different degrees of increase in classification accuracy. The Adaboost algorithm can be used for the prediction of diabetes follow-up and control satisfaction data.

## 1. Introduction

Currently, China has the highest number of chronic disease patients in the world, of which those suffering from diabetes and its associated complications are among the most critical. Diabetes is a chronic disease characterized by a long treatment cycle, numerous complications (e.g., kidney and eye diseases), and recurrent illness. With advances in the informatization of medicine, medical industries with large amounts of complicated patient data are keen to extract information from this data to assist the development of these industries. Simultaneously, they also seek to be capable of alleviating the challenges faced by medical personnel, through the forthcoming development of smart medicine. The use of machine learning and other artificial intelligence methods for the analysis of medical data in order to assist diagnosis and treatment is one of the manifestations of smart medicine with the most practical significance.

With the improvement of the living standards of our people and the westernization of our diet, the incidence, mortality, and morbidity of diabetes have significantly increased and have a serious impact on our health. In 2006, Shang [1] made use of the survey data of Xinjiang chronic disease integrated prevention and control demonstration site in the New Urban District of Urumqi in 2004 and surveyed 2031 people over the age of 18 in three communities in the district. The results showed the relationship between diabetes and age and gender: the prevalence of male and female rose with age, because the decrease of glucose tolerance with age and the improvement of living standard are the reasons for the increased incidence. Overweight and obesity are one of the risk factors of diabetes mellitus. The survey found that the prevalence of diabetes in people with BMI>24 was 10. 58%, the prevalence of diabetes in people with BMI≦24 was 4.31%, two groups prevalence by chi-square test was P <0.01, and there was a significant difference between the two groups, indicating that overweight and obese individuals are more susceptible to diabetes. In 2009, Su [2] analyzed the related factors of diabetes in the New Urban District of Urumqi in Xinjiang. The results showed that age, gender, height, weight, and BMI associated with diabetes were not statistically significant. However, the waist circumference, systolic blood pressure, and triglyceride are factors that are positively correlated with diabetes. In 2017, Mohemaiti [3] used questionnaire to survey the prevalence of 200 elderly patients type 2 diabetes with coronary heart disease from January to December in 2016 in Hangzhou Road community of the New Urban Area of Urumqi; the results showed that smoking, BMI ≥ 24 kg/m^2^, complications associated with diabetes, hypertension, and dyslipidemia are risk factors for coronary heart disease in elderly patients with diabetes mellitus. It is the key according to the relevant risk factors and the timely development of interventions to reduce the prevalence of coronary heart disease in elderly patients with diabetes mellitus.

Data mining is a significant tool in medical databases, which enhances the sensitivity and/or specificity of disease detection and diagnosis by opening a window of relatively better resources [4]. Applying machine learning and data mining methods in diabetes research is a pivotal way to utilizing plentiful available diabetes-related data for extracting knowledge. The severe social impact of the specific disease makes DM one of the main priorities in medical science research, which inevitably produces large amounts of data. Therefore, there is no doubt that machine learning and data mining approaches in DM are of great concern on diagnosis, management, and other related clinical administration aspects [5]. In order to achieve the best classification accuracy, abundant algorithms and diverse approaches have been applied, such as traditional machine learning algorithms, ensemble learning approaches, and association rule learning. Most noted among the aforementioned ones are the following: Calisir and Dogantekin proposed LDA-MWSVM, a system for diabetes diagnosis [6]. The system performs feature extraction and reduction using the Linear Discriminant Analysis (LDA) method, followed by classification using the Morlet Wavelet Support Vector Machine (MWSVM) classifier. Gangji and Abadeh [7] presented an Ant Colony-based classification system to extract a set of fuzzy rules, named FCSANTMINER, for diabetes diagnosis. In [8], authors regard glucose prediction as a multivariate regression problem utilizing Support Vector Regression (SVR). Agarwal [9] utilized semi-automatically marked training sets to create phenotype models via machine learning methods. Ensemble approaches, which utilize multiple learning algorithms, have been confirmed to be an effective way of enhancing classification accuracy.

This study follows the support vector machine (SVM), Adaboost, Bagging data mining ensemble techniques, and decision tree as our research model. More specifically, the dataset used for decision-making in this study is obtained from the diabetes follow-up data of the New Urban Area of Urumqi, Xinjiang. The purpose of this study is to evaluate the performance of aforementioned techniques of data mining and adopt machine learning methods that combine feature selection and class unbalanced processing to evaluate the health management control satisfaction of diabetic patients. We used health management measure indicators of diabetes patients as the input variables of our models to accurately classify two levels of control satisfaction in follow-up data, namely, (i) satisfied with the control and (ii) unsatisfied with the control. Finally, a classification model with further higher classification accuracy was constructed.

## 2. Materials and Methods

### 2.1. Dataset

The dataset used in this study is gathered from the diabetic patient health management follow-up data of the New Urban Area of Urumqi, Xinjiang. The dataset contains 3406 records for a period ranging from December 1, 2016, to February 28, 2017. Each record includes 25 characteristic variables, which are likely to affect the degree of satisfaction with diabetes control. An abstract detail of those relevant factors selected in this study is provided in Table 1 that includes age, sex, race, body mass index (BMI), diabetes complications, systolic blood pressure, diastolic blood pressure, and fasting blood glucose of the patients. The chi-square test was used to compare and analyze the satisfaction of different classification variables and the respondents. By using chi-square test to select a small number of the most relevant features (or by eliminating many irrelevant features), one is able to reduce the risk of overfitting the training data and often produce a better overall model. The difference was statistically significant at P<0.05. Categorical variables are statistically significant by chi-square test and continuous variables, which are used as input variables for machine learning.

In our research, the dataset encounters the class imbalance problem. Out of 3406 patients, 2832 patients were satisfied with control of diabetes, which constitutes about 83.21% of the total patients and 574 patients are unsatisfied. The imbalanced ratio equals 5:1 between majority and minority. In other words, a dataset is class-imbalanced if one class includes significantly more sample numbers than the other. In order to resolve the problem, we can pick the random undersampling (RUS), random oversampling (ROS), and SMOTE, which are among the most used resampling methods to counterpoise imbalanced datasets. Here, we only choose SMOTE algorithms, which is used to create one more dataset, where the minority samples were oversampled by 400% and the majority class was undersampled at 123% to approximately make the ratio 1:1. The descriptions of the datasets are given in Table 2. Eventually, the balanced dataset was used to construct the model.

### 2.2. Algorithms

We selected 4 algorithms to test decision tree, support vector machine (SVM), Bagging, and Adaboost which are common algorithms in machine learning. Decision tree [10] is a category of tree classifier. Generally, decision tree uses information entropy, information gain, or Gini coefficients to assess which characteristic to use as the classification characteristic corresponding to a non-leaf-node [11]. Ordinarily, decision trees can intuitively display the classification process, clearly showing rules that can be understood by humans. SVMs are supervised learning models associated with data analysis and model recognition and are widely used in classification and regression analysis, which use a hypothesis space of polynomial linear functions over a high dimensional feature space. While SVMs are a “black box” algorithm, they typically outperform other ML algorithms for classification tasks [12, 13]. In 1996, Breiman proposed the popular bootstrap aggregation (Bagging) method [14]. It primarily involves bootstrap sampling techniques in which samples are selected repeatedly with a certain probability and with replacement, which generates numerous different sample subsets. Next, these different sample subsets are used individually to perform training on base classifiers and obtain an integrated classifier with certain diversity. The diversity strategy of Bagging is straightforward and effective, and numerous derivative methods based on this strategy yield adequate classification results [15]. Boosting, also known as reinforcement learning, is a critical ensemble learning technique that can reinforce a weak classifier, whose prediction accuracy is marginally higher than that of a random guess, into a strong classifier with high prediction accuracy. Adaboost is the most successful representative of this algorithm and has been rated as one of the ten most effective algorithms for data mining [16]. This algorithm is an iterative method that was proposed by Schapire and Freund in 1995 [17–19].

Because each of these algorithms has their own characteristics and advantages, each method will produce different results to classify the degree of satisfaction of diabetes follow-up and control, and for more comprehensive evaluation of predictors in the imbalanced context, G-mean [20] and AUC [21] are frequently used to measure how well the predictor can balance the performance between two classes, so we choose G-mean and area under the ROC curve (AUC) as an index to evaluate the performance of the classification models. By using confusion matrix (see Table 3), we can calculate the accuracy, sensitivity, and specificity.

G-mean is the geometric mean of the sensitivity and specificity; that is,

(1) $$\begin{array}{c} G\text{-}mean=\sqrt{Sensitivity\times Specificity} \end{array}$$

The ROC curve describes the relationship between TP/(TP + FN) and FP/(FP + TN) of the classifier. Since the ROC curve cannot quantitatively evaluate the classifiers, AUC is usually adopted as the evaluation index. AUC (area under ROC curve) value refers to the area under the ROC curve. An ideal classification model has an AUC value of 1, with a value between 0.5 and 1.0, and the larger AUC represents that the classification model has better performance.

The experimentation is performed using open source R software version 3.4.1 (https://www.r-project.org/). The main packages included the following:

(1) The adabag (https://cran.r-project.org/web/packages/adabag/) software package focuses on the Bagging and Adaboost algorithms.

(2) The kernlab (https://cran.r-project.org/web/packages/kernlab/) package was used for the support vector machine algorithm.

(3) The rpart (https://cran.r-project.org/web/packages/rpart/) was used for decision tree classification.

## 3. Results

**Figure 1:**
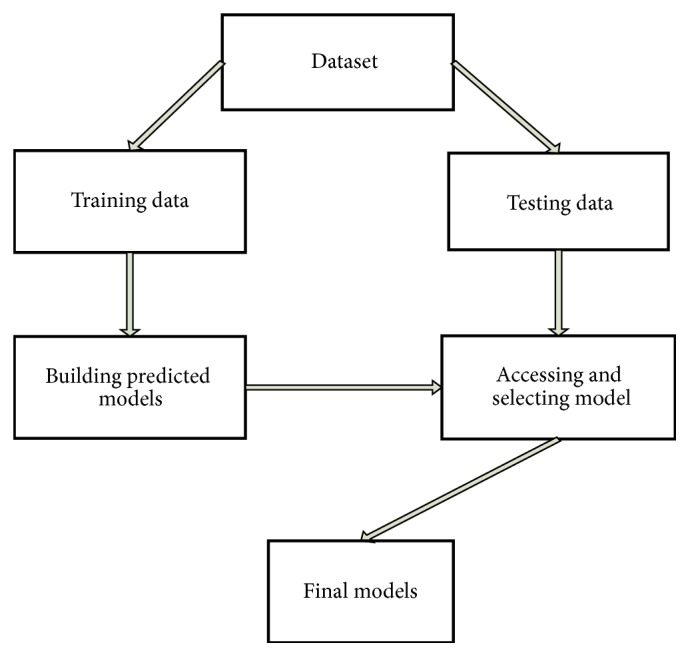
General flowchart of modeling.

Our research dataset is divided into two parts; two-thirds of the data is used as a training set, and one-third of the dataset is defined as a testing set to evaluate the performance of several classifiers. All classifiers were fitted to the same training and testing data. The specific process is shown in Figure 1.

**Figure 2:**
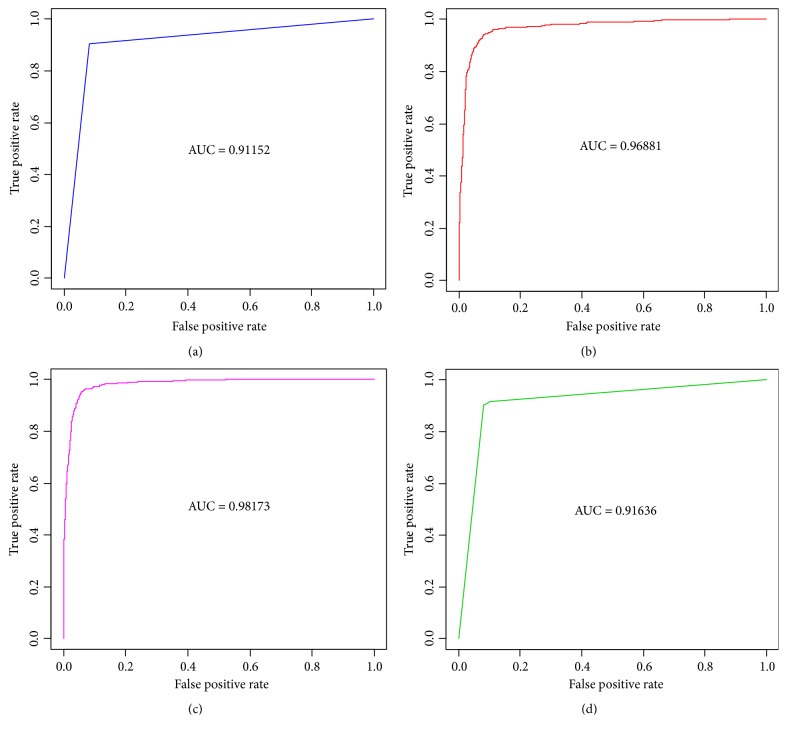
ROC curves for (a) decision tree model, (b) SVM model, (c) Adaboost model, and (d) Bagging model.

As can be seen from Table 4, in this study, the performance of the four final predictive models was evaluated using G-mean, AUC. For the testing dataset, the final comparative analysis results demonstrated that the Adaboost algorithm showed the best with accuracy of 94.84%, and the sensitivity and specificity were 95.76% and 93.56%, respectively. The SVM algorithm came out to be the second best with a classification accuracy of 92.62%, and the sensitivity and specificity gave 94.08% and 91.28%, respectively, followed by the Bagging model (91.15%) and decision tree (91.15%), which exhibited identical results, with the sensitivity and specificity being equal to 90.50% and 91.81%, respectively. In the results, the area under the receiver operating characteristic (ROC) curve (AUC) values of the SVM, Bagging, and decision tree algorithms were 0.9688, 0.9164, and 0.9115, respectively. The area under ROC for Adaboost ensemble method is 98.17% and G-mean of 0.9465, showing a high reliability of discriminative capability among all the methods. Overall, the ML method presented in this paper has obtained the well classification performance of health management control satisfaction of patients with diabetes. Decision tree also yielded better performance. The ROC curves for the four classifiers are shown in Figure 2.

## 4. Discussion

Health management of diabetic patients is an important part of the national basic public health service project. Diabetics are one of the six key groups defined by the national basic public health service project, and satisfaction is one of the important indicators of the effectiveness of the test project [22]. Patients are satisfied with the services provided; they will take the initiative to participate in the project to form a virtuous circle, further enhance the effectiveness of project health management, and then promote the smooth implementation of the project. At the same time, patient satisfaction with health services directly affects the development of health services. Therefore, we must attach great importance to the satisfaction of patients and improve patient satisfaction by continuously improving service capabilities and service quality [23]. Machine learning methods provide a new way to diabetes analytics which is suitable for contemporary Big Data demands. Those approaches could get over many constraints intrinsic in many traditional statistical modeling approaches [24]. Therefore, when focusing on a certain disease, several appropriate classification algorithms should be selected based on the characteristics of the dataset. By comparing the classification accuracy of these classification algorithms on the dataset, the most effective classification algorithm is used as the diagnostic model. In general, the performance of machine learning algorithms is evaluated using predictive accuracy. However, this is not appropriate when the data is imbalanced and/or the costs of different errors vary markedly.

The dataset used in this study is obtained from the diabetic patient health management follow-up data of the New Urban Area of Urumqi, Xinjiang. This study systematically involves four representative data mining techniques for predictive data mining task. That includes decision tree, SVM, ensemble learning method Bagging, and Adaboost. These algorithms are combined for creating knowledge to render it useful for decision-making. Each algorithm will produce different results to classify the degree of satisfaction with diabetes control. Firstly, chi-square test was used to select the features of the diabetes dataset. Secondly, because the dataset has unbalanced problem, we chose a method to deal with unbalanced data, that is, the SMOTE method. Finally, the dataset after feature selecting and unbalanced processing was classified by four classification algorithms. The experimental results proved that, for the testing dataset, Adaboost algorithm performed best in four models with a AUC equal to 0.9817 and an G-mean equal to 0.9465. An important feature of the Adaboost algorithm is the calculation of the importance of each variable (feature). We can output the importance score of each input variable in the classification process. Variables with high importance are closely related to the predictions results. For instance, Huang [25] mentioned that adequately controlled blood glucose was defined as fasting blood glucose values <7.0 mmol/L. The effect of post-management blood glucose control has a direct impact on patient satisfaction, with a statistically significant difference (X2=24.128, P<0.05). Moreover, Baccaro [26] also indicated that a significant statistic correlation was observed between the score of the questionnaires and good diabetes control showed by the levels of HbAc1 and fasting blood glucose, among other parameters, which is consistent with the first important variable (fasting blood glucose) reported by the Adaboost algorithm proposed by us. Our results also showed that the age and BMI were also important variables. One study has pointed out [27] higher age, better physical health, less diabetes-related distress, and higher diabetes treatment satisfaction. Another example, a previous study [28] aims to assess the psychological well-being and treatment satisfaction in patients with type 2 diabetes mellitus in a general hospital in Korea. Their result revealed that treatment satisfaction was significantly associated with age, satisfaction with waiting and treatment times, compliance with recommended diet and exercise, and duration of diabetes. For BMI, there is a certain relationship between the satisfaction rate of blood glucose control and overweight or obesity, which explains the importance of BMI in the classification of control satisfaction [29]. Besides, to determine which patient characteristics and laboratory values were independently associated with treatment satisfaction, Boels [30] used a linear mixed model for analysis, whose conclusion was that a number of factors including diabetes education, perceived and actual hyperglycaemia, and macrovascular complications are associated with treatment satisfaction. The Bagging and Adaboost methods [31] combine a large number of decision trees and can significantly increase their prediction efficiency. Ensemble learning algorithm has better performance than simple classification algorithm (decision tree).

The limitations of research should also be recognized. In this paper, only one method of dealing with unbalanced data is used. Of course, all kinds of methods have been developed to deal with unbalanced data, such as random oversampling, cluster-based oversampling, and algorithmic ensemble techniques. This paper does not compare with the performance of the original dataset in the algorithm. In the future work, we can consider, from a variety of perspectives, adopting diverse imbalanced processing methods and a machine learning method to compare the effects of different types of unbalanced processing techniques.

In addition, it should be referred that despite the claims that these machine learning classification algorithms can generate sufficient and effective decision-making, very few have really permeated the clinical practice [32]. Understandably, clinicians are not only interested in the high accuracy of a predictive model, but also in the degree with which the model could explain the pathogenesis of the disease [24]. Although it has powerful learning capabilities, without being supported by the appropriate approaches for determining how they work, the results of machine learning algorithms prediction may encounter a limited applicability in the clinical practices. We used machine learning approaches for diabetes analytics in real-life clinical settings, which is a severe challenge.

## 5. Conclusions

In this study, we used the diabetic patient health management follow-up data. We have combined feature selection and imbalanced processing techniques, and few researchers have utilized the health management control satisfaction of patients with diabetes for classification predictions. In this work, we offered proof that Adaboost algorithm can be successfully used for health management control satisfaction of patients with diabetes.

## Figures and Tables

**Table 1:** Analysis of control satisfaction of diabetes patients in New Urban Area of Urumqi (n=3406).

**Characteristic**	**Satisfied** **(N1=574)**	**Unsatisfied** **(N2=2832)**	**χ** ^2^	***P* values**
**Age, Median (IQR), Years**	57(49-65)	54(46-62)	-	-
**Sex**				
male	276	1400	0.35	0.555
female	298	1432		
**Ethnicity**				
Han nationality	479	2544	28.05	*<0.0001*
Hui	57	183		
others	3	28		
Uighur	35	77		
**Degree of education**				
junior high school	193	866	12.62	*0.013*
College specialties and above	55	392		
High School / Technical School	96	559		
Illiteracy and semi-literacy	56	245		
primary school	174	770		
**Marital status**				
Divorced / widowed	59	362	2.79	0.248
unmarried	3	13		
married	512	2457		
**Diagnosis methods**				
clinical	228	1673	73.96	*<0.0001*
outpatient clinic	333	1099		
others	13	60		
**Diabetes complications**				
Coronary heart disease				
no	525	2462	9.07	*0.003*
yes	49	370		
Hypertension				
no	311	1317	11.27	*0.001*
yes	263	1515		
High cholesterol				
no	483	2579	25.17	*<0.0001*
yes	91	253		
**Smoking**				
no	270	1546	10.94	*0.001*
yes	304	1286		
**Drinking**				
no	278	1622	15.13	*<0.0001*
yes	296	1210		
**Diet control**				
no	187	666	20.88	*<0.0001*
yes	387	2166		
**physical activities**				
no	158	621	8.48	*0.004*
yes	416	2211		
**Hypoglycemic agents**				
no	175	802	1.10	0.295
yes	399	2030		
**Insulin**				
no	337	1722	0.88	0.349
yes	237	1110		
**Quit smoking**				
no	356	1903	5.72	*0.017*
yes	218	929		
**Limit wine**				
no	333	1863	12.58	*<0.0001*
yes	241	969		
**Follow-up method**				
phone	50	218	9.75	*0.008*
home	26	234		
clinic	498	2380		
**Psychological adjustment**				
poor	8	13	78.86	*<0.0001*
good	327	2123		
fair	239	696		
**Follow medical practice**				
poor	98	103	191.40	*<0.0001*
good	254	1863		
fair	222	866		
**Compliance medication**				
no medication	80	421	41.89	*<0.0001*
regular	455	2356		
intermittent	39	55		
**Systolic blood pressure, Median (IQR), mm Hg**	130 (120-140)	130 (120-140)	-	-
**Diastolic blood pressure, Median (IQR), mm Hg**	78 (70-80)	80 (70-84)	-	-
**BMI, Median (IQR), kg/m** ^**2**^	25.36 (23.53-27.53)	26.27 (24.14-28.43)	-	-
**Fasting blood glucose level, Median (IQR), mmol/L**	6.4 (6.0-6.8)	8.7 (7.5-11.03)	-	-

**Table 2:** Dataset description.

Dataset	Samples distribution	Ratio	Description
Original data	2832/574	5:1	Original data with full instances

SMOTE-data	2824/2870	1:1	Dataset is balanced utilizing SMOTE oversampling

**Table 3:** Confusion matrix.

		Predicted classification	
		1	0
Actual classification	1	TP	FP
	0	FN	TN

**Table 4:** Comparison of prediction performance of the four models.

Algorithms	Accuracy	Sensitivity	Specificity	G-mean	AUC
Decision Trees	0.9115	0.9050	0.9181	0.9115	0.9115
SVM	0.9262	0.9408	0.9128	0.9267	0.9688
Adaboost	0.9484	0.9576	0.9356	0.9465	0.9817
Bagging	0.9115	0.9050	0.9181	0.9115	0.9164

## Data Availability

The data used to support the findings of this study are available from the corresponding author upon request.

## References

[1] Shang X. J., Liu L. X., Guan Y. M. (pp. 69-69.2006). Analysis of the results of diabetes investigation in the New Urban Area of Urumqi, Xinjiang. *Bulletin of Disease Control and Prevention*.

[2] Su L. Q., Wang F. P., Wang X. Y. (2009). Analysis of related factors of diabetes in the New Urban Area of Urumqi, Xinjiang. *Xinjiang Medicine*.

[3] Mohemaiti P., Keyoumu Y., Mohemaiti P. (2017). Current situation and related risk factors of elderly type 2 diabetes mellitus with coronary heart disease in Hangzhou road community of the New Urban Area of Urumqi. *Chinese Journal of Gerontology*.

[4] Perveen S., Shahbaz M., Guergachi A. (2016). Performance Analysis of Data Mining Classification Techniques to Predict Diabetes. *Procedia Computer Science*.

[5] Kavakiotis I., Tsave O., Salifoglou A., Maglaveras N., Vlahavas I., Chouvarda I. (2017). Machine Learning and Data Mining Methods in Diabetes Research. *Computational and Structural Biotechnology Journal*.

[6] Çalişir D., Doğantekin E. (2011). An automatic diabetes diagnosis system based on LDA-wavelet support vector machine classifier. *Expert Systems with Applications*.

[7] Ganji M. F., Abadeh M. S. (2011). A fuzzy classification system based on ant colony optimization for diabetes disease diagnosis. *Expert Systems with Applications*.

[8] Georga E. I., Protopappas V. C., Ardigò D. (2013). Multivariate Prediction of Subcutaneous Glucose Concentration in Type 1 Diabetes Patients Based on Support Vector Regression. *IEEE Journal of Biomedical Health Informatics*.

[9] Agarwal V., Podchiyska T., Banda J. M. (2016). Learning statistical models of phenotypes using noisy labeled training data. *Journal of the American Medical Informatics Association *.

[10] Rokach L., Maimon Z. O. (2008). *Data mining with decision trees: theory and applications*.

[11] Rokach L., Maimon O. (2005). Top-down induction of decision trees classifiers - a survey. *IEEE Transactions on Systems Man & Cybernetics Part C*.

[12] Cossock D., Zhang T. (2008). Statistical analysis of Bayes optimal subset ranking. *Institute of Electrical and Electronics Engineers Transactions on Information Theory*.

[13] Mani S., Chen Y., Elasy T. (2012). Type 2 diabetes risk forecasting from EMR data using machine learning. *AMIA Annual Symposium Proceedings*.

[14] Breiman L. (1996). Bagging predictors. *Machine Learning*.

[15] Breiman L. (2001). Random forests. *Machine Learning*.

[16] Zhou Z. H., Yang Y., Wu X. D., Kumar V. (2009). *The Top Ten Algorithms in Data Mining*.

[17] Freund Y., Schapire R. E. (1997). A decision-theoretic generalization of on-line learning and an application to boosting. *Journal of Computer and System Sciences*.

[18] Freund Y., Schapire E. R. Experiments with a new boosting algorithm.

[19] Schapire R. E., Singer Y. (1999). Improved boosting algorithms using confidence-rated predictions. *Machine Learning*.

[20] Kubat M., Matwin S. Addressing the curse of imbalanced training sets: One-sided selection.

[21] Bradley A. P. (1997). The use of the area under the ROC curve in the evaluation of machine learning algorithms. *Pattern Recognition*.

[22] Meng F. L., JIN S. H. (2012). Investigation and Analysis of Satisfaction of Hypertension Patients in Community Health Service in Hangzhou. *Health Research*.

[23] Zhao Y. F., Yao X., Deng F. (2015). Survey and Analysis of the Satisfaction of the Residents of the Community Health Service Institutions in Karamay City. *Chinese Journal of Social Medicine*.

[24] Sebastian Y. (2017). Advances in Diabetes Analytics from Clinical and Machine Learning Perspectives. *International Journal of Design, Analysis and Tools for Integrated Circuits and Systems*.

[25] Huang L., Liu Y., Guan X. J. (2017). Satisfaction of Diabetic Patients under Community Health Management in Wuhou District of Chengdu, 2014-2016. *Journal of Preventive Medicine Information*.

[26] Baccaro F., Novelli P. P., Arduin J. (2016). Diabetes Treatment Satisfaction Questionnaire (DTSQ) of in non-ambulatory type 2 diabetic patients. *Boletín De La Asociación Médica De Puerto Rico*.

[27] Wermeling P. R., Janssen J., Gorter K. J., Beulens J. W. J., Rutten G. E. H. M. (2013). Satisfaction of well-controlled type 2 diabetes patients with three-monthly and six-monthly monitoring. *BMC Family Practice*.

[28] Park H., Lee S. N., Baek M. Y. (2016). The Well-Being and Treatment Satisfaction of Diabetic Patients in an Outpatient Setting at a General Hospital in Korea. *The Journal of Korean Diabetes*.

[29] Xie H. J. (2013). Investigation and Analysis of Blood Glucose Control. *The Chinese community physicians (medicine)*.

[30] Boels A. M., Vos R. C., Hermans T. G. (2017). What determines treatment satisfaction of patients with type 2 diabetes on insulin therapy? An observational study in eight European countries. *BMJ Open*.

[31] Mani S., Chen Y., Elasy T. (2012). Type 2 Diabetes Risk Forecasting from EMR Data using Machine Learning. *AMIA Annual Symposium Proceedings*.

[32] Kourou K., Exarchos T. P., Exarchos K. P., Karamouzis M. V., Fotiadis D. I. (2015). Machine learning applications in cancer prognosis and prediction. *Computational and Structural Biotechnology Journal*.

